# Good and bad outcomes of respiratory viral infections—influenza A virus trains sustained antitumor immunity of macrophages in the lung

**DOI:** 10.1038/s41423-023-01015-y

**Published:** 2023-04-17

**Authors:** Sabine Stegemann-Koniszewski, Sarah Frentzel, Dunja Bruder

**Affiliations:** 1 Experimental Pneumology, Department of Pneumology, University Hospital Magdeburg/Medical Faculty, Health Campus Immunology, Infectiology and Inflammation, Otto-von-Guericke University Magdeburg, Magdeburg, Germany; 2 Institute of Medical Microbiology and Hospital Hygiene, Infection Immunology Group, Health Campus Immunology, Infectiology and Inflammation, Otto-von-Guericke University Magdeburg, Magdeburg, Germany; 3 Immune Regulation Group, Helmholtz Centre for Infection Research, Braunschweig, Germany

**Keywords:** Lung cancer, Alveolar macrophages

In their recent article in Nature Immunology, Wang et al. showed that influenza A virus (IAV) infection trained alveolar macrophages (AMs) for sustained antitumor immunity in the lung [1], extending the concept of trained immunity and pointing out benefits of viral respiratory infection in the context of pulmonary antitumor immune surveillance.

Trained immunity describes the enhanced responsiveness of innate myeloid cells to secondary stimuli following exposure to a transient primary trigger. It involves transcriptional, epigenetic, and metabolic changes and can be local or systemic, with the latter effect leading to changes in bone marrow progenitors [2]. Little is known about the role of trained immunity in antitumor responses, and whether sustained antitumor functions are achievable in tissue-resident macrophages is unclear.

**Fig. 1:**
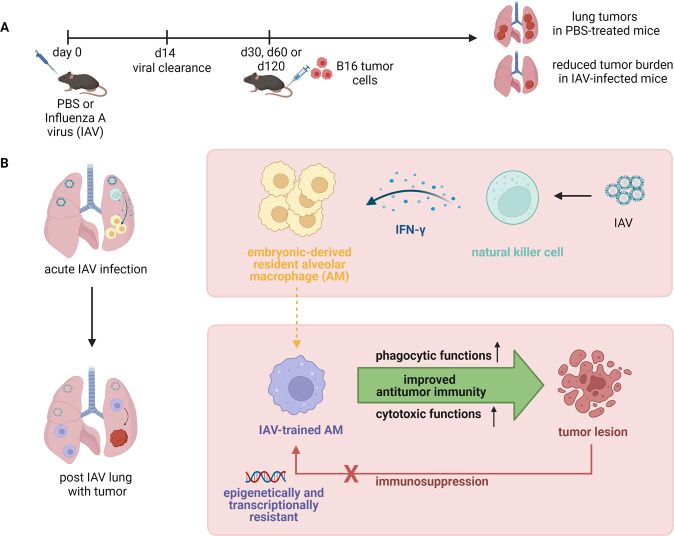
Acute IAV infection induces long-lasting antitumor immunity in the lungs (**A**). Natural killer cell-derived interferon (IFN)-γ trains antitumor effector functions in embryo-derived resident alveolar macrophages and renders them resistant to tumor-induced immune suppression (**B**). The figure was created using BioRender.com

The authors showed that in a mouse model of sublethal IAV infection and injection of B16 melanoma cells 30 days later, IAV infection induced pulmonary antitumor immunity. The infected mice showed a significantly reduced lung tumor burden and increased survival after tumor challenge (Fig. 1A). A consistent antitumor phenotype was observed when B16 cells were inoculated 60 or 120 days post-IAV infection as well as following adenovirus (AdV) infection. These antitumor effects were not observed after subcutaneous injection of B16 cells, but in spontaneous lung metastases in a breast cancer model. Furthermore, the effects were independent of changes to hematopoietic cells in the bone marrow, showing that respiratory viral infection triggered long-term antitumor immunity specifically in the lung.

Depletion of T cells or AMs and adoptive transfer of virus-exposed AMs to naïve recipients showed that IAV-induced antitumor immunity was independent of adaptive T cells but depended on virus-exposed AMs. Analyses of the transcriptional profile, phenotype, immune functions, and chromatin accessibility in AMs 30 days following IAV infection revealed cardinal features of trained immunity: profoundly altered gene transcription, increased major histocompatibility complex class II expression, increased proinflammatory cytokine secretion after stimulation, sustained metabolic rewiring and increased chromatin accessibility in genes related to immune activation and macrophage effector functions. Using different approaches, the authors showed that IAV-trained AMs developed independent of circulating monocytes. An analysis of the proliferation status of the AMs at different time points after infection suggested that despite substantial losses during acute infection, the AMs were replenished via the proliferation of embryonic-derived resident AMs, which then induced tissue-specific trained immunity.

To identify the mechanism by which IAV-trained AMs exert antitumor immunity, the authors employed a number of approaches. Abundant AMs were detected in juxtaposition to B16 cells in tumor lesions, and higher rates of tumor cell phagocytosis by IAV-trained AMs than by IAV-naïve AMs were observed. Upregulated transcription of gene clusters closely related to macrophage antitumor functions identified in RNA-seq data obtained from IAV-trained AMs supported the hypothesis suggesting that AMs promote antitumor immunity through enhanced phagocytosis and tumoricidal functions. In vitro coculture experiments demonstrated significantly decreased survival of B16 cells in the presence of AMs from IAV-infected mice compared to those from PBS-treated mice. This difference was not observed when cells were separated by transwell membranes, indicating contact-dependent mechanisms. Flow cytometry and imaging of cocultured cells confirmed phagocytosis of tumor cells by IAV-trained AMs. The killing of phagocytosed cells was independent of AM death, but the degree of tumoricidal functions was reduced when mitochondrial oxidation was suppressed. This outcome suggested that AMs depend on enhanced mitochondrial oxidation from fatty acids and glucose. Tumor-associated macrophages (TAMs) derived from tissue-resident macrophages harbor the potential for both protumor and antitumor functions and are important to oncogenesis [3]. Immunosuppression of TAMs is a key mechanism in tumor cell immune escape. For functional characterization of IAV-trained AMs within the tumor microenvironment, peritumoral AMs were purified after the establishment of B16 cell lung tumors and subjected to ATAC-seq and RNA-seq analyses. The resulting data suggested that tumor progression induced the establishment of an immune-suppressive microenvironment to which IAV-trained AMs were epigenetically and transcriptionally resistant (Fig. 1B).

Prompted by findings from AdV-mediated trained immunity [4], the authors asked whether IAV-mediated trained immunity depends on T cells and/or interferon (IFN)-γ. When CD4^+^ and CD8^+^ T cells were depleted from day 3 post-infection until inoculation with B16 cells, a reduced tumor burden was nevertheless evident in IAV-infected mice. Analyses in IFN-γ and IFN-γ receptor knockout mice showed that IAV-mediated antitumor immunity depended on IFN-γ. Natural killer (NK) cells are significant sources of early IFN-γ following IAV infection [5]. Depletion of T cells or NK cells and analysis of antitumor functions in the lungs revealed that NK cells, but not T cells, were critical for IAV-induced antitumor AM functions.

To determine whether their observations applied to human lung cancer, the authors sought to identify trained AMs in human lung tumors and determine whether they are associated with antitumor immunity. Analysis of published single-cell RNA-seq data from non-small cell lung cancer tissues led to the identification of multiple AM subsets, one of which harbored transcriptional profiles characteristic of trained immunity. These trained AMs were enriched with relevant gene clusters, and the frequency of these cells was significantly increased in lung cancer tissues with high immune activation compared with those with low immune activation in the tumor microenvironment.

This study presents intriguing new aspects of trained immunity. In a broader context, the observations contribute to evidence suggesting the significance of multiple, sequential triggers for tissue immune homeostasis and emphasize the thin line between the good and bad outcomes of respiratory microbial infections. IAV remains a serious potentially life-threatening pathogen that predisposes patients to severe bacterial pneumonia [6]. Also in this study, mice were highly susceptible to *Streptococcus pneumoniae* during acute IAV infection, but they showed increased survival and reduced bacterial burden after a lethal pneumococcal challenge 30 days post-infection. Ultimately, detailed mechanistic knowledge is essential to exploit protective mechanisms. As the lung represents a preferential site for primary and metastatic tumors with unfavorable prognoses, there is a clear need for novel therapeutic strategies [7]. Since IAV-trained antitumor immunity prolonged survival by only a few days, the induction of sustained AM antitumor activity might not represent an ultimate cure. Nevertheless, it is a promising contributor to successful antitumor immunity in the lung.
